# The Influence of Maternal Vitamin D Supplementation in Pregnancies Associated with Preeclampsia: A Case-Control Study

**DOI:** 10.3390/nu14153008

**Published:** 2022-07-22

**Authors:** George Dahma, Radu Neamtu, Razvan Nitu, Adrian Gluhovschi, Felix Bratosin, Mirela Loredana Grigoras, Carmen Silaghi, Cosmin Citu, Igwe Nwobueze Orlu, Sanket Bhattarai, Adelina Geanina Mocanu, Marius Craina, Elena Bernad

**Affiliations:** 1 Department of Obstetrics and Gynecology, “Victor Babes” University of Medicine and Pharmacy Timisoara, 300041 Timisoara, Romania; george_dahma@yahoo.com (G.D.); radu.neamtu@umft.ro (R.N.); adigluhovschi@yahoo.com (A.G.); silaghi.carmen@gmail.com (C.S.); citu.ioan@umft.ro (C.C.); adelinaerimescu@yahoo.com (A.G.M.); mariuscraina@hotmail.com (M.C.); ebernad@yahoo.com (E.B.); 2 Methodological and Infectious Diseases Research Center, Department of Infectious Diseases, “Victor Babes” University of Medicine and Pharmacy Timisoara, 300041 Timisoara, Romania; felix.bratosin7@gmail.com (F.B.); grigoras.mirela@umft.ro (M.L.G.); 3 Faculty of General Medicine, University of Debrecen Medical School, Nagyerdei Street 94, 4032 Debrecen, Hungary; igweorlu@gmail.com; 4 KIST Medical College, Imadol Marg, Lalitpur 44700, Nepal; dr.sanketnep@gmail.com

**Keywords:** preeclampsia, risk factors, vitamin D, pregnancy

## Abstract

Preeclampsia is a pregnancy-specific illness that is hypothesized to occur due to vitamin D deficiency during pregnancy. Therefore, vitamin D supplementation in early pregnancy should be explored for preventing preeclampsia and promoting neonatal well-being. The present study follows a case-control analysis that aims to determine the effect of vitamin D supplements on reducing the probability of recurrent preeclampsia. We identified 59 patients for the control group without vitamin D supplementation during pregnancy, while 139 patients were included in the cases group of pregnant women with a history of preeclampsia who confirmed taking daily vitamin D supplements in either 2000 UI or 4000 UI until the 36th week of pregnancy. There were 61 (80.3%) patients with a normal serum vitamin D level measured at 32 weeks in the pregnant women who took a daily dose of 4000 UI vitamin D and 43 (68.3%) in those who took a 2000 UI dose of vitamin D, compared to just 32 (54.2%) in those who did not take vitamin D at all. Regarding the blood pressure of pregnant women measured at 32 weeks, it was observed that 20.3% were hypertensive in the no supplementation group, compared to only 11.1% and 6.6% in those who were taking vitamin D during pregnancy (*p*-value = 0.049). Serum vitamin D levels at 32 weeks were measured at an average value of 23.9 ng/mL, compared with 28.4 ng/mL in the group taking a 2000 UI daily dose and 33.6 in those who supplemented with 4000 UI daily (*p*-value < 0.001). Proteinuria was identified more often in the group at risk for preeclampsia who did not take vitamin D supplements, while systolic blood pressure (*p*-value = 0.036) as well as diastolic blood pressure (*p*-value = 0.012), were all identified to have significantly higher values in the pregnant women with a history of preeclampsia that did not take vitamin D during the current pregnancy. The significant risk factors for preeclampsia development in pregnant patients at risk are: insufficient vitamin D serum levels (<20 ng/mL), OR = 2.52; no vitamin D supplementation, OR = 1.46; more than two pregnancies, OR = 1.89; gestational diabetes mellitus, OR = 1.66; and cardiovascular comorbidities, OR = 2.18. These findings imply that vitamin D has a role in the preservation of placental function and, therefore, in the prevention of the development of late preeclampsia. Pregnant mothers who supplemented their diets with vitamin D were protected against preeclampsia recurrence. Vitamin D supplementation during pregnancy may aid in the prevention of gestational hypertension and preeclampsia.

## 1. Introduction

Preeclampsia is a pregnancy-specific illness defined by proteinuria and high blood pressure developed after the first twenty weeks of pregnancy while causing a quarter of all maternal fatalities and perinatal morbidity and mortality. Existing data shows that preeclampsia complicates between 2–10% of all pregnancies, with an average of 4–5% worldwide [1]. Although preeclampsia involves more than simple gestational hypertension with proteinuria, the development of proteinuria remains a major objective diagnostic indicator for this condition. Proteinuria is characterized by measuring 300 mg or more of protein in a 24-h urine collection, a protein-creatinine ratio of 0.3 or more in random urine samples, or a constant quantity of protein in random urine samples [2].

Vitamin D insufficiency in pregnant women, as well as other multiple deficiencies that frequently happen during pregnancy, is a common public health concern [3,4]. Recent studies discovered that almost 30% of African-American pregnant women and 5%of Caucasian pregnant women residing in the northeastern United States had abnormal vitamin D levels consistent with vitamin D deficiency, defined as serum 25-hydroxyvitamin D levels of less than 37.5 nmol/L, whereas 54% of African-American women, and, respectively, 47% of Caucasian women had serum 25(OH)D levels indicative of vitamin D insufficiency [5].

Throughout pregnancy, hypocalciuria and low vitamin D levels have been repeatedly reported in women who ultimately developed preeclampsia [6]. Vitamin D insufficiency is sometimes considered a global pandemic, as it can range from 18-84%, varying by geographical factors, ethnicity, local dressing norms, and culinary traditions [7,8]. Vitamin D3 is a micronutrient that is created endogenously when UV-B is exposed to the skin and was discovered to have a function in bone disorders and calcium metabolism [9]. After its conversion to 1,25-dihydroxy vitamin D3 (1,25(OH)D), which is the high-affinity ligand of the nuclear transcription factor vitamin D receptor, vitamin D3 influences gene regulation (VDR). In the proximity of its target genes, ligand-activated VDR binds to accessible genomic locations and controls their transcription, with potential multi-organ effects [10]. By regulating the expression of important associated-developmental genes, it is now well acknowledged that vitamin D has a function in several organs, including the placenta, as, during pregnancy, the concentrations of 1,25(OH)D in the maternal systemic circulation and the placenta tend to have a physiological rise [11].

It is considered that diabetes, chronic hypertension before pregnancy, chronic renal illnesses, nulliparity, twin or multiple pregnancies, familial history of preeclampsia or eclampsia, obesity, immunological problems, and a personal history of preeclampsia or eclampsia are risk factors for preeclampsia. Preeclampsia in a single pregnancy is not always predictive of its development in later pregnancies, but its first occurrence is connected with a greater likelihood that it will recur in later pregnancies [12]. Maternal vitamin D insufficiency may lead to issues such as low birth weight and small-for-gestational-age children, in addition to the increased risk of maternal comorbidities [13]. Clinical investigations investigating the association of low vitamin D and unfavorable pregnancy outcomes that comprise preeclampsia, gestational diabetes, low birth weight, premature labor, and cesarean section, have contradictory findings [14].

Low vitamin D levels alter the equilibrium between Th1 and Th2 and lead to the overexpression of Th1 cytokines, according to previous research. The latter event impacts embryo implantation’s immune tolerance. The data indicate that a deficit in vitamin D may be connected with the increased expression of Th1 seen in preeclampsia [15]. As one of the possibilities of the cause of preeclampsia is a deficit in serum 25(OH)D levels during pregnancy, it was considered in the current research that pregnant women having a history of preeclampsia in prior pregnancies to be analyzed in terms of vitamin D serum levels and vitamin D supplementation. Given that one of the suspected causes of preeclampsia is an increased demand for vitamin D during pregnancy giving a vitamin D supplement will likely satisfy this increased need and enable a proper evaluation of its function in avoiding preeclampsia. Therefore, the purpose of this study was to retrospectively evaluate the association between preeclampsia in pregnant women at risk, and their nutritional supplementation during pregnancy. It was hypothesized that higher doses of vitamin D supplementation and higher serum levels of 25(OH)D would carry a lower risk of developing preeclampsia.

## 2. Materials and Methods

### 2.1. Study Design and Inclusion Criteria

In cooperation with the “Victor Babes” University of Medicine and Pharmacy, an observational single-centric case-control study was developed in a retrospective fashion between 2018 and 2022 in the Department of Obstetrics and Gynecology from the Timis County Emergency Hospital “Pius Brinzeu”. The study utilized a population-based database of inpatients and outpatients of the same clinic over the course of four years. The research cohort included pregnant women with a history of preeclampsia and their associated features that were documented from the administrative database of the hospital. Patient cases were considered those with vitamin D supplementation during pregnancy, while the control group comprised those who did not take vitamin D supplements during pregnancy. The centralized database of patients comprised their records including demographic data, medical history, and in-hospital procedures, which were gathered with the patient’s consent and protected by existent privacy laws. All patients’ initial characteristics and treatment regimens were documented in the hospital database and in paper patient records that qualified clinicians involved in the current investigation reviewed. A computerized database search was performed to identify the precise diagnosis as specified by the Current Procedural Terminology and the International Classification of Diseases.

Pregnant women at risk for preeclampsia were considered eligible for inclusion in the current study based on the guidelines’ definition of preeclampsia according to the American College of Obstetrics and Gynecology (ACOG) [16]. Therefore, all pregnant women who were identified after 20 weeks of gestation with a systolic blood pressure of 140 mm Hg or higher or a diastolic blood pressure of 90 mm Hg or higher, at two separate times that are at least four hours apart or at a lower period timing of systolic blood pressure of 160 mm Hg or higher, or a diastolic blood pressure of 110 mm Hg or higher. In addition to the high blood pressure values, another criterion is the presence of proteinuria or other signs of organ damage unrelated to other preexistent conditions [17]. Regarding the existence of a personal medical history of preeclampsia and signs indicating preeclampsia in a previous pregnancy, it was considered that pregnant women in a second pregnancy are at risk for preeclampsia, and careful monitoring and management should be considered. Patients were not included in the research if their medical records were found to be lacking important data or if the permission form was not properly completed and included with the patient’s previous paperwork.

### 2.2. Ethical Considerations

The Timis County Emergency Clinical Hospital “Pius Brinzeu” Local Commission of Ethics for Scientific Research operates in accordance with Article 167 of Law No. 95/2006, article 28, Chapter VIII of order 904/2006, with EU GCP Directives 2005/28/EC, the International Conference on Harmonisation of Technical Requirements for Registration of Pharmaceuticals for Human Use (ICH), and with the Declaration of Helsinki regarding the Recommendations Guiding Medical Practice. In addition, the Commission adheres to all of the current investigation protocols, and was given approval on 20 April 2022, with the number 46.

### 2.3. Study Variables

The variables considered for statistical analysis comprised maternal background data (age, gravidity, parity, area of residence, occupation, level of education, level of income, civil status); pregnancy-associated conditions (gestational diabetes mellitus, abnormal presentation, premature rupture of membranes, anemia, peripartum infections, other maternal infections); comorbidities (cardiovascular, respiratory, digestive, autoimmune, others); other supplements taken during pregnancy (calcium and magnesium, folate, iron, probiotics); neonatal characteristics comprising the gender, APGAR score, birth weight, in-vitro fertilization, type of delivery, infection after membrane rupture, congenital abnormalities, prematurity, neonatal intensive care unit admission (NICU), resuscitation, days of hospitalization, days of NICU stay, mortality, and therapy with surfactant, steroids, and antibiotics. Patient records were measured at 32 and 36 weeks, comprising vitamin D deficiency severity, presence of hypertension, serum levels of active vitamin D, systolic and diastolic blood pressure. Lastly, maternal risk factors for preeclampsia were identified. According to the World Health Organization, a peripartum infection is a bacterial infection of the genital tract or surrounding tissues that can occur at any time between the beginning of the rupture of the membranes or labor and the 42nd day after giving birth. Peripartum infections are most common in women who have given birth recently [18]. Patients’ records were screened to determine the type of nutritional supplementation taken during pregnancy and the dose taken, as well as the new cases of preeclampsia in these patients. Serum levels of maternal vitamin D were determined by immunochemical method with electrochemiluminescence detection, measuring total vitamin D (25(OH)D). A value below 30 ng/mL was considered as vitamin D insufficiency.

### 2.4. Statistical Analysis

For statistical analysis, the IBM SPSS software version 27.0 (Armonk, NY, USA: IBM Corp.) and MedCalc (MedCalc Software, Ostend, Belgium) statistical analysis software were employed. Categorical variables were represented using absolute and percentage values. The ANOVA and the Mann-Whitney U-test were used for parametric and non-parametric variables, respectively. To determine the normality of the data, the Shapiro-Wilk test was used. The proportions were statistically examined using the Chi2 and Fisher’s exact tests. After controlling for confounding factors, a multivariate regression analysis was carried out using a stepwise method to analyze independent risk variables for developing preeclampsia. To assess the likelihood of developing preeclampsia for these categories, a Kaplan-Meier probability curve was produced for independent risk variables. The significance criteria were set at the 0.05 threshold.

## 3. Results

A total of three study groups were created based on vitamin D supplementation during the pregnancy period. A number of 59 pregnant women with a history of preeclampsia did not take vitamin D during the second pregnancy, as recommended by the physician, while a group of 63 admitted to taking a daily dose of 2000 units of vitamin D during the first trimester. Lastly, the third group comprising 76 pregnant women admitted taking 4000 UI of vitamin D oral supplementation.

### 3.1. Maternal Background Analysis

Table 1 presents the comparison of maternal background characteristics stratified by the amount of vitamin D supplement during the first trimester. It was observed that more than 60% of all patients were in the 25 to 34 years age range, without significant differences between study groups. The majority of patients had one child from a previous pregnancy, in a proportion of 69.3%. Other background characteristics of the study participants identified a prevalence of approximately 60% of patients coming from an urban living area, and more than 80% of them were currently employed. There was no significant difference in the level of education, level of income, and civil status of the pregnant women included in the current study.

The pregnancy characteristics stratified by type of vitamin D oral supplementation during pregnancy are presented in Table 2. It was observed that anemia was the most common finding in these patients, with an average of 35% among all three groups, followed by an abnormal fetal presentation in approximately 9% of cases. Gestational diabetes mellitus was also observed in 15 (7.5%) of all study participants, which is an important finding as it is considered a known pregnancy-associated risk factor for preeclampsia. The most prevalent comorbidity was obesity in 7 (11.9%) of pregnant women who did not supplement with vitamin D during the current pregnancy, 12 (19.0%) in the low-dose vitamin D group, and 10 (13.2%) in the high-dose group (*p*-value = 0.478). The second most common comorbidity was cardiovascular disease, identified in 10 (5.0%) of all patients, without significant changes among the study groups (*p*-value = 0.752). The third most common comorbidity was digestive conditions, followed by respiratory. Regarding the nutritional supplementation during pregnancy, the study participants were taking calcium and magnesium, folate, iron, and probiotics, without statistically significant differences between groups. The most common supplement taken was folate by 165 (83.3%) of all patients, followed by iron supplements and calcium/magnesium by a third of all patients.

### 3.2. Neonatal and Pregnancy Outcomes

The neonatal characteristics and pregnancy outcomes presented in Table 3 did not show significant differences between groups, except for premature births and the need for antibiotic treatment of the newborn. There were 8 (13.6%) cases of prematurity in the group of pregnancies without vitamin D supplementation, compared to just 2 (3.2%) and, respectively, 2 (2.6%) in the groups of vitamin D supplementation (*p*-value = 0.030). Similarly, significant differences were observed between neonate patients in the no vitamin D supplementation group compared to the low dose and high dose supplementation groups, where 18 (30.5%) required antibiotic treatment during hospital stay after birth, compared to only 12 (19.0%) and 10 (13.2%) in the vitamin D supplementation groups (*p*-value = 0.043). However, there were no significant findings when comparing the pregnancy outcomes between the pregnant women who took 2000 UI of vitamin D and those who took 4000 UI.

### 3.3. Analysis at 32 and 36 Weeks of Pregnancy

**Figure 1:**
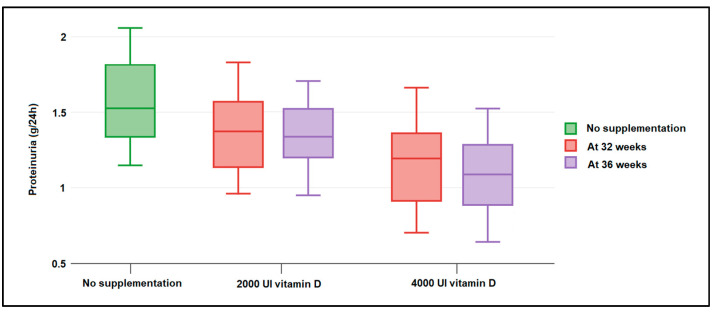
Boxplot of maternal proteinuria (g/L) measured at 32 and 36 weeks of gestation and stratified by nutritional supplementation with 2000 UI and 4000 UI of vitamin D. Data analyzed by Kruskal–Wallis test.

**Figure 2:**
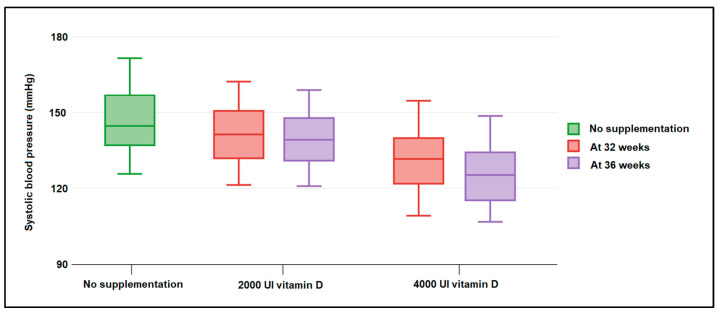
Boxplot of maternal systolic blood pressure (mm Hg) measured at 32 and 36 weeks of gestation and stratified by nutritional supplementation with 2000 UI and 4000 UI of vitamin D. Data analyzed by Kruskal–Wallis test.

The patient records at 32 weeks and 36 weeks were collected and analyzed in Table 4. It was observed that a significantly higher proportion of moderate and severe vitamin D deficiency was documented in pregnant women who did not take vitamin D supplements during pregnancy (20.3% in the no supplementation group vs. 12.7% in the low vitamin D supplementation group, respectively, 6.6% in the high dose supplementation group, *p*-value = 0.027). Therefore, there were 61 (80.3%) patients with a normal serum vitamin D level measured at 32 weeks in the pregnant women who took a daily dose of 4000 UI vitamin D and 43 (68.3%) in those who took a 2000 UI dose of vitamin D, compared to just 32 (54.2%) in those who did not take vitamin D at all. Regarding the blood pressure of pregnant women measured at 32 weeks, it was observed that 20.3% were hypertensive in the no supplementation group, compared to only 11.1% and 6.6% in those who were taking vitamin D during pregnancy (*p*-value = 0.049). Serum vitamin D levels at 32 weeks were measured at an average value of 23.9 ng/mL, compared with 28.4 ng/mL in the group taking a 2000 UI daily dose and 33.6 in those who supplemented with 4000 UI daily (*p*-value < 0.001). Proteinuria was identified more often in the group at risk for preeclampsia who did not take vitamin D supplements, as seen in Figure 1. Systolic blood pressure (*p*-value = 0.036), as well as diastolic blood pressure (*p*-value = 0.012), were all identified to have significantly higher values in pregnant women with a history of preeclampsia that did not take vitamin D during the current pregnancy (Figure 2). Similar findings were observed in the same variables measured at 36 weeks, with a slight improvement in those who took vitamin D, regardless of the dose.

### 3.4. Risk Analysis

**Figure 3:**
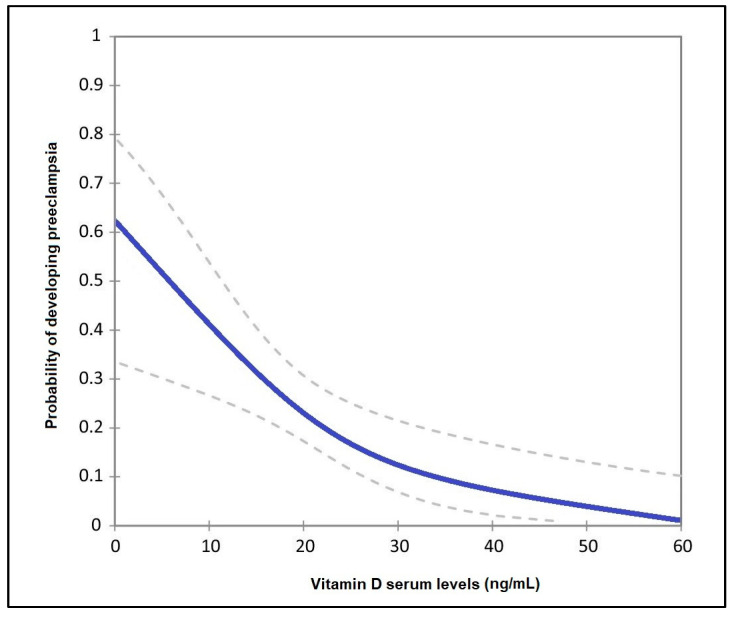
Adjusted probability of developing preeclampsia by levels of vitamin D.

The risk analysis presented in Table 5 identified a statistically significant association between vitamin D serum levels and the likelihood of developing preeclampsia in the second pregnancy. Therefore, pregnant patients with insufficient (<20 ng/mL) serum vitamin D showed a 2.52 higher likelihood of preeclampsia (95% CI = 1.86–3.90), no vitamin D supplementation patients had a 1.46 higher likelihood (95% CI = 1.12–1.86), having a history of more than two pregnancies carried a risk of 1.89 (*p*-value = 0.008), gestational diabetes mellitus carried a risk level for preeclampsia of 1.66 (*p*-value = 0.017). Lastly, cardiovascular comorbidities carried a 2.18 higher likelihood of developing preeclampsia (*p*-value = 0.001). The adjusted probability of developing preeclampsia in the studied population is presented in Figure 3.

## 4. Discussion

### 4.1. Supporting Literature

Based on the current findings and existing literature, it can be admitted that nutritional supplementation of vitamin D during the first 32 weeks of pregnancy can reduce the recurrence of preeclampsia among pregnant women at-risk, and improve other pregnancy outcomes such as the occurrence of high blood pressure. Insufficient vitamin D levels was an independent risk factor for preeclampsia, and pregnant patients with vitamin D insufficiency were observed to have a 2.5 times higher likelihood of placenta-mediated problems. There was a dose-response association between maternal 25(OH) D levels at 32 weeks and the later risk of pregnancy-associated hypertension and preeclampsia.

The supporting literature regarding vitamin D’s impact on preeclampsia suggests that vitamin D metabolism is connected with preeclampsia through several physiological pathways through which the vitamin D level of the mother might influence the risk of preeclampsia in two phases [19]. In the first stage of pregnancy, placental perfusion is diminished, which in association with abnormal implantation can cause a further diminution of blood supply. Poor placental blood flow can generate the secretion of hormones and several blood regulators, which in a favorable maternal environment begin the subsequent multisystem disorder that represents phase two of preeclampsia development. However, it seems that inadequate placental blood flow is not the primary cause of preeclampsia, although it is a potent risk factor [20]. Furthermore, trophoblastic immaturity was observed to be associated with low vitamin D levels and its receptor involved in trophoblastic syncytization [21,22].

Endothelial dysfunction is another important determining factor in preeclampsia being part of a systemic intravascular inflammatory response involving leukocytes, coagulation, and complement systems that can be modified by levels of maternal vitamin D. It seems that renal vascular endothelial growth factor (VEGF) is related with proteinuria present in preeclampsia, while 1,25-dihydroxyvitamin D3 may be able to influence angiogenic processes via influencing VEGF gene transcription [23]. It has also been proven that the active form of vitamin D, 1,25-dihydroxyvitamin D3, modifies the transcription and activity of genes involved in proper implantation, placental invasion, and angiogenesis [24]. The immunomodulatory characteristics of 1,25-dihydroxy vitamin D suggest that aberrant implantation is possible and mediated by an inappropriate immunological response between the expectant mother and fetus [25,26].

Pregnant women often suffer from vitamin D insufficiency, as observed by the low levels of 25-hydroxyvitamin D [25(OH) D] determined by the participants in the current study. Other authors found a very high incidence of vitamin D insufficiency, with 78% of all studied cases having a 25(OH) D level under 30 ng/mL [27]. Here the normal pregnancies had a mean serum 25(OH) D level of 24.86 ng/mL, whereas preeclamptic women had a mean serum 25(OH) D level of 23.96 ng/mL, and eclamptic women had a mean serum 25(OH) D level of 21.56 ng/mL. Among pregnant women with vitamin D deficiency, the odds ratios for developing preeclampsia and eclampsia were 3.9 and 5.14, respectively, when adjusted for age, BMI, and length of pregnancy [28].

One meta-analysis of observational studies [29] showed a significant connection between vitamin D levels and poor pregnancy outcomes such as premature delivery, gestational diabetes mellitus, and preeclampsia, the latter showing a substantial connection between vitamin D insufficiency and preeclampsia, with a 4.2 higher likelihood than pregnant women without a history of preeclampsia [30]. Another recent meta-analysis showed a link between vitamin D and preeclampsia in a variety of study designs, demonstrating that vitamin D may have a role in preventing preeclampsia [31]. For example, pregnant women received a multivitamin and mineral supplement, including halibut liver oil providing approximately 900 units of vitamin D from 20 weeks of gestation, which determined a reduction in the risk of preeclampsia by 32% [32]. A large randomized trial comprising 400 women treated with a daily intake of 1200 units of vitamin D plus a calcium supplement at 20–24 weeks of pregnancy had a significant reduction in blood pressure but a nonsignificant reduction in the incidence of preeclampsia in the group at risk compared to the placebo group [33].

The majority of research has documented vitamin D profiles in the general population but not in high-risk groups. At 14 weeks, patients with preeclampsia had reduced vitamin D levels. At 20 ng/mL, the dose-effect association between vitamin D concentration and pre-eclampsia risk had doubled, while a rise in 25(OH)D concentration of at least 12 ng/mL was a protective factor regardless of low levels measured in the first trimester [34]. In addition, pregnant women with adequate vitamin D levels throughout the third trimester and both the first and third trimesters had a considerably reduced chance of developing preeclampsia [35]. Other research examined vitamin D levels throughout the second trimester only, and it was observed that before 22 weeks, vitamin D insufficiency was a substantial and independent risk factor for preeclampsia, as well as a dose-response relationship existing between the levels measured before 22 weeks of gestation. Overall, a concentration of less than 20 ng/mL may double the chance of developing preeclampsia. Despite vitamin supplementation, these findings were seen in 93% of patients three months before giving birth and in 46% of patients throughout the periconceptional period [29,36]. In addition, pregnant women who later developed severe pre-eclampsia had a lower 25(OH)D concentration between 18 and 20 weeks [37].

Another research found no significant difference in 25(OH)D concentrations between those with and without preeclampsia, despite the fact that 80% of patients had vitamin D insufficiency [38]. However, the majority of studies emphasized the link between vitamin D insufficiency and preeclampsia, which seemed to be most prominent between 32 and 36 weeks of pregnancy in high-risk groups [39]. Lastly, an important evidence according to the most recent meta-analysis suggests that insufficient maternal 25(OH)D serum levels may raise the risk of preeclampsia [40].

### 4.2. Study Strengths and Limitations

The current study is among few research to address the history of preeclampsia as a factor of vitamin D levels in pregnant mothers. Although the current research managed to determine important findings regarding maternal vitamin D levels in pregnancies at-risk for preeclampsia, this study has several limitations worth mentioning. Being a monocentric study, only patients from a single site were tested for vitamin D, leaving for certain bias risks and small sample size. In addition, the research did not evaluate other factors of vitamin D concentrations, such as seasonality or fetal blood sampling. The retrospective design also limits our findings, as well as the manual data collection from patient records, does increase the risk of error.

## 5. Conclusions

Pregnant women after 32 weeks of gestation with vitamin D insufficiency have an increased risk of placenta-mediated hypertension or preeclampsia. These findings imply a connection between vitamin D status and the preservation of placental function and, thus, the avoidance of preeclampsia. Vitamin D is a feasible treatment for the prevention of preeclampsia, and well-controlled, randomized trials are required immediately to confirm its effectiveness and safety.

## Figures and Tables

**Table 1:** Background data of the study cohort stratified by type of vitamin D oral supplementation.

	No Supplementation (*n* = 59)	Low Dose (*n* = 63)	High Dose (*n* = 76)	*p*-Value *
Age				0.255
<25	12 (20.3%)	14 (22.2%)	11 (14.5%)	
25–34	38 (64.4%)	44 (69.8%)	49 (64.5%)	
>34	9 (15.3%)	5 (7.9%)	16 (21.1%)	
Gravidity				0.288
2	50 (84.7%)	48 (76.2%)	56 (73.7%)	
>2	9 (15.3%)	15 (23.8%)	20 (26.3%)	
Parity				0.931
1	39 (66.1%)	46 (73.0%)	53 (69.7%)	
2	11 (18.6%)	10 (15.9%)	14 (18.4%)	
≥3	9 (15.3%)	7 (11.1%)	9 (11.8%)	
Area of residence				0.958
Urban	35 (59.3%)	38 (60.3%)	44 (57.9%)	
Rural	24 (40.7%)	25 (39.7%)	32 (42.1%)	
Occupation				0.662
No occupation	4 (6.8%)	7 (11.1%)	11 (14.5%)	
Student	4 (6.8%)	6 (9.5%)	6 (7.9%)	
Employed	51 (86.4%)	50 (79.4%)	59 (77.6%)	
Level of education				0.292
Elementary	3 (5.1%)	6 (9.5%)	10 (13.2%)	
Middle	18 (30.5%)	12 (19.0%)	14 (18.4%)	
Higher	39 66.1%)	45 (71.4%)	52 (68.4%)	
Level of income				0.963
Low	6 (10.2%)	6 (9.5%)	8 (10.5%)	
Middle	33 (55.9%)	38 (60.3%)	41 (53.9%)	
High	20 (33.9%)	19 (30.2%)	27 (35.5%)	
Civil status				0.937
Married	54 (91.5%)	57 (90.5%)	68 (89.5%)	
Single	2 (3.4%)	3 (4.8%)	5 (6.6%)	
Divorced/Widowed	3 (5.1%)	3 (4.8%)	3 (3.9%)	

* Chi-square or Fisher’s exact test.

**Table 2:** Pregnancy characteristics are stratified by the type of vitamin D oral supplementation during pregnancy.

	No Supplementation (*n* = 59)	Low Dose (*n* = 63)	High Dose (*n* = 76)	*p*-Value *
History of pregnancy-associated conditions				
Gestational diabetes mellitus	4 (6.8%)	4 (6.3%)	7 (9.2%)	0.787
Abnormal presentation	6 (10.2%)	5 (7.9%)	7 (9.2%)	0.911
PROM	4 (6.8%)	6 (9.5%)	4 (5.3%)	0.618
Anemia	23 (39.0%)	24 (38.1%)	26 (34.2%)	0.825
Peripartum infection	3 (5.1%)	5 (7.9%)	4 (5.3%)	0.750
Other maternal infections	3 (5.1%)	2 (3.2%)	6 (7.9%)	0.472
Comorbidities				
Cardiovascular	2 (3.4%)	4 (6.3%)	4 (5.3%)	0.752
Obesity **	7 (11.9%)	12 (19.0%)	10 (13.2%)	0.478
Respiratory	1 (1.7%)	4 (6.3%)	3 (3.9%)	0.426
Digestive	3 (5.1%)	1 (1.6%)	3 (3.9%)	0.561
Autoimmune	0 (0.0%)	1 (1.6%)	2 (2.6%)	0.461
Others	2 (3.4%)	2 (3.2%)	5 (6.6%)	0.554
Other supplements taken				
Calcium/Magnesium	14 (23.7%)	19 (30.2%)	25 (32.9%)	0.501
Folate	45 (76.3%)	51 (81.0%)	69 (90.8%)	0.066
Iron	20 (33.9%)	31 (49.2%)	38 (50.0%)	0.125
Probiotics	12 (20.3%)	22 (34.9%)	27 (35.5%)	0.114

* Chi-square or Fisher’s exact test; ** Calculated in correlation with gestational age.

**Table 3:** Neonatal characteristics are stratified by maternal vitamin D oral supplementation during pregnancy.

	No Supplementation (*n* = 59)	Low Dose (*n* = 63)	High Dose (*n* = 76)	*p*-Value *
Neonatal characteristics				
Gender (male)	33 (55.9%)	32 (50.8%)	36 (47.4%)	0.613
Abnormal APGAR score	5 (8.5%)	4 (6.3%)	4 (5.3%)	0.753
Birth weight *** (grams), mean ± SD	2731 ± 552	2880 ± 594	2926 ± 518	0.117
Conceived by vitro fertilization	1 (1.7%)	0 (0.0%)	1 (1.3%)	0.609
Delivered by C-section	28 (47.5%)	33 (52.4%)	30 (39.5%)	0.303
Infection after membrane rupture	5 (8.5%)	7 (11.1%)	7 (9.2%)	0.875
Congenital abnormalities	2 (3.4%)	2 (3.2%)	1 (1.3%)	0.691
Prematurity	8 (13.6%)	3 (4.8%)	2 (2.6%)	0.030
NICU admission	2 (3.4%)	1 (1.6%)	1 (1.3%)	0.667
Resuscitation	4 (6.8%)	2 (3.2%)	4 (5.3%)	0.657
Days of hospitalization **	4 (3)	4 (2)	3 (2)	0.492
Therapy				
Surfactant	5 (8.5%)	2 (3.2%)	2 (2.6%)	0.221
Steroids	6 (10.2%)	4 (6.3%)	4 (5.3%)	0.524
Antibiotics	18 (30.5%)	12 (19.0%)	10 (13.2%)	0.043

* Chi-square or Fisher’s exact test; ** Data represented as median [IQR]; *** In correlation with gestational age; APGAR—Appearance, Pulse, Grimace, Activity, Respiration; NICU—Neonatal Intensive Care Unit.

**Table 4:** Patient records at 32 weeks and 36 weeks of gestation.

	No Supplementation (*n* = 59)	Low Dose (*n* = 63)	High Dose (*n* = 76)	*p*-Value *
At 32 weeks				
Low vitamin D (<30 ng/mL)				0.027
Insufficient < 20 ng/mL (*n* = 25)	12 (20.3%)	8 (12.7%)	5 (6.6%)	
Vitamin D deficiency 20–30 ng/mL (*n* = 37)	15 (25.4%)	12 (19.0%)	10 (13.2%)	
Normal serum vitamin D >30 ng/mL (*n* = 136)	32 (54.2%)	43 (68.3%)	61 (80.3%)	
Hypertension				0.049
Hypertensive	12 (20.3%)	7 (11.1%)	5 (6.6%)	
Non-hypertensive	47 (79.7%)	56 (88.9%)	71 (93.4%)	
Serum vitamin D, ng/mL (mean ± SD)	23.9 ± 7.3	28.4 ± 8.0	33.6 ± 7.1	<0.001
Systolic blood pressure (mean ± SD)	139.4 ± 33.1	130.2 ± 26.6	127.4 ± 22.5	0.036
Dyastolic blood pressure (mean ± SD)	85.2 ± 14.6	80.4 ± 11.6	79.1 ± 10.2	0.012
At 36 weeks				
Vitamin D deficiency (<30)				0.006
Insufficient < 20 ng/mL (*n* = 25)	14 (23.7%)	7 (11.1%)	4 (5.3%)	
Vitamin Ddeficiency 20–30 g/mL (*n* = 47)	17 (28.8%)	15 (23.8%)	15 (19.7%)	
Normal serum vitamin D >30 ng/mL (*n* = 126)	28 (47.5%)	41 (65.1%)	57 (75.0%)	
Hypertension				0.002
Hypertensive	15 (25.4%)	7 (11.1%)	4 (5.3%)	
Non-hypertensive	10 (74.6%)	22 (88.9%)	20 (94.7%)	
Serum vitamin D, ng/mL (mean ± SD)	22.5 ± 8.1	29.1 ± 7.7	35.6 ± 8.3	<0.001
Systolic blood pressure (mean ± SD)	141.4 ± 38.9	129.4 ± 30.5	127.3 ± 28.7	0.034
Dyastolic blood pressure (mean ± SD)	86.4 ± 15.9	81.2 ± 10.6	80.5 ± 9.4	0.012
Preeclampsia	11 (18.6%)	6 (9.5%)	4 (5.3%)	0.041

* Chi-square or Fisher’s exact test; Data represented as mean ± SD; SD—Standard Deviation.

**Table 5:** Maternal risk factor analysis for preeclampsia.

Risk Factors	OR	95% CI	*p*-Value
Insufficient vitamin D serum levels (<20 ng/mL)	2.52	1.86–3.90	<0.001
No vitamin D supplementation	1.46	1.12–1.86	0.042
Parity > 2	1.89	1.42–2.31	0.008
Gestational diabetes mellitus	1.66	1.09–2.24	0.017
Cardiovascular comorbidities	2.18	1.58–2.93	0.001

## Data Availability

The data presented in this study are available on request from the corresponding author.
